# A case of relapsed acute myeloid leukemia mimicking acute otomastoiditis

**DOI:** 10.1002/ccr3.8717

**Published:** 2024-03-26

**Authors:** Ivan Negara, Inga Chemencedji, Natalia Dobrovolschi, Natalia Sporis, Sanda Buruiana, Igor Vinogradov

**Affiliations:** ^1^ “Nicolae Testemitanu” State University of Medicine and Pharmacy Chisinau Moldova; ^2^ Institute of Oncology Chisinau Moldova

**Keywords:** acute myeloid leukemia, hematology, mastoiditis, myeloid sarcoma, oncology, relapse

## Abstract

**Key Clinical Message:**

Identifying myeloid sarcoma in rare locations is a diagnostic challenge and requires careful evaluation. The optimal management of extramedullary disease requires further investigation, but tissue biopsy and a personalized approach are crucial.

**Abstract:**

Herein, we describe an unusual case of acute myeloid leukemia presenting with an isolated involvement of the temporal bone after a complete remission of systemic disease for more than a year. The clinical, radiological, and pathological features are discussed, highlighting the importance of considering differential diagnoses and appropriate management.

## Graphical abstract

A computed tomography scan reveals a complete homogenous left‐sided and partial right‐sided opacification of the mastoid cells and the middle ear with a relatively intact bony structure and no distinct masses.

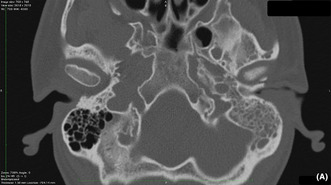

## 1 INTRODUCTION

Acute myeloid leukemia (AML) is a heterogenous hematologic malignancy characterized by clonal proliferation of immature myeloid cells.^1^ A rare form of AML is myeloid sarcoma (MS), which manifests as an extramedullary collection of myeloid tumor cells.^2^ It can present concurrently with systemic disease or, rarely, as the sole manifestation before systemic involvement.^2^ In the latter case, it may take months before the disease manifests in the blood or the bone marrow, leading to significant diagnostic challenges and delayed recognition. Although myeloid sarcomas can develop in various tissues and organs,^3^, ^4^ there is limited data on extramedullary AML affecting the head and neck region.^5^ Here, we describe a rare otologic case of an adult relapsed AML patient with an isolated temporal bone involvement.

## 2 CASE HISTORY

**FIGURE 1:**
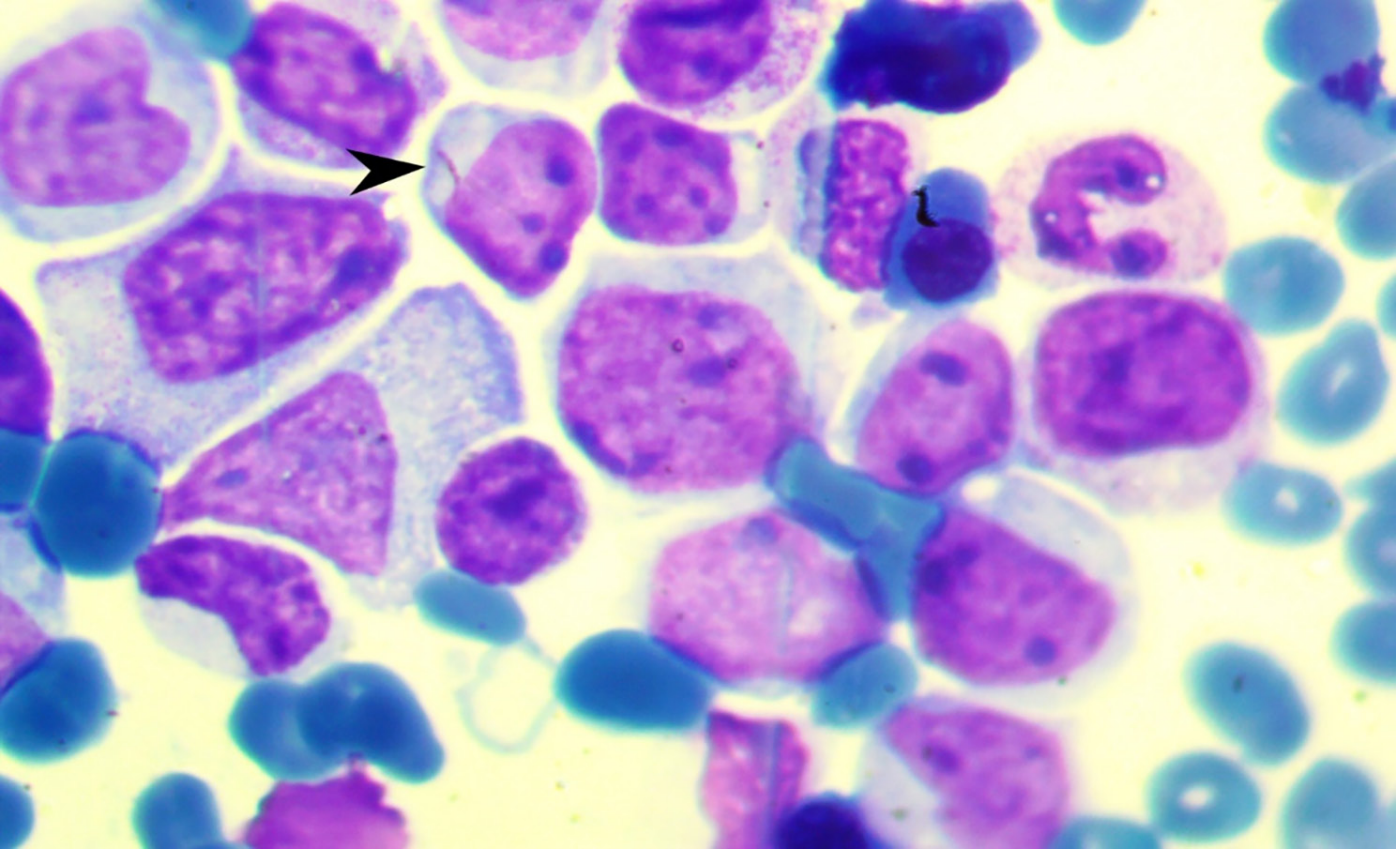
Bone marrow aspirate. Bone marrow examination reveals the presence of myeloblasts. A characteristic Auer rod (arrowhead) can be seen.

A 32‐year‐old male without any previous medical history initially presented with a 1‐month history of progressive weakness, dyspnea, and intermittent fever. Physical examination revealed petechiae on the upper extremities and thorax. The peripheral blood cell count showed the following: white blood cell count (WBC), 75.8 × 10^9^/L with 42% blast cells, hemoglobin level of 7.2 g/dL, and a platelet count of 54 × 10^9^/L. Subsequent bone marrow aspiration revealed a hypercellular bone marrow with a significant increase in cells displaying morphological features consistent with myeloblasts (Figure 1). A diagnosis of AML, subtype M4 (acute myelomonocytic leukemia)  was established.

## 3 INVESTIGATIONS AND TREATMENT

Standard induction chemotherapy was initiated with an anthracycline‐based 7 + 3 regimen containing doxorubicin and cytarabine. After the first cycle, repeat bone marrow aspiration showed no blast cells, and the complete blood count (CBC) revealed a hemoglobin level of 10.6 g/dL, platelet count of 204 × 10^9^/L, and WBC count of 6 × 10^9^/L with a normal differential, indicating complete remission with hematologic recovery. The cerebrospinal fluid (CSF) analysis was unremarkable for signs of disease.

Post‐remission therapy consisted of three additional consolidation cycles of the same regimen, followed by monthly surveillance. After approximately 16 months of complete remission (CR), the patient presented to a local otolaryngology department with complaints of left‐sided otalgia, hearing loss, and fever. Otoscopic examination revealed a narrowed left external auditory canal with ipsilateral hyperemia and purulent otorrhea obscuring the tympanic membrane. Examination of the right side was unremarkable. Bilateral mixed hearing loss was confirmed by audiometry. The CBC results did not show any signs of systemic AML, except for a slight leukocytosis with a left‐shift deviation suggestive of an inflammatory process. Likewise, the CSF examination indicated no evidence of leukemic cells. Based on presentation and symptoms, a presumptive diagnosis of acute otitis media and externa was made, and the patient was given oral antibacterial therapy. However, after 1 week of treatment, there was no symptomatic improvement.

**FIGURE 2:**
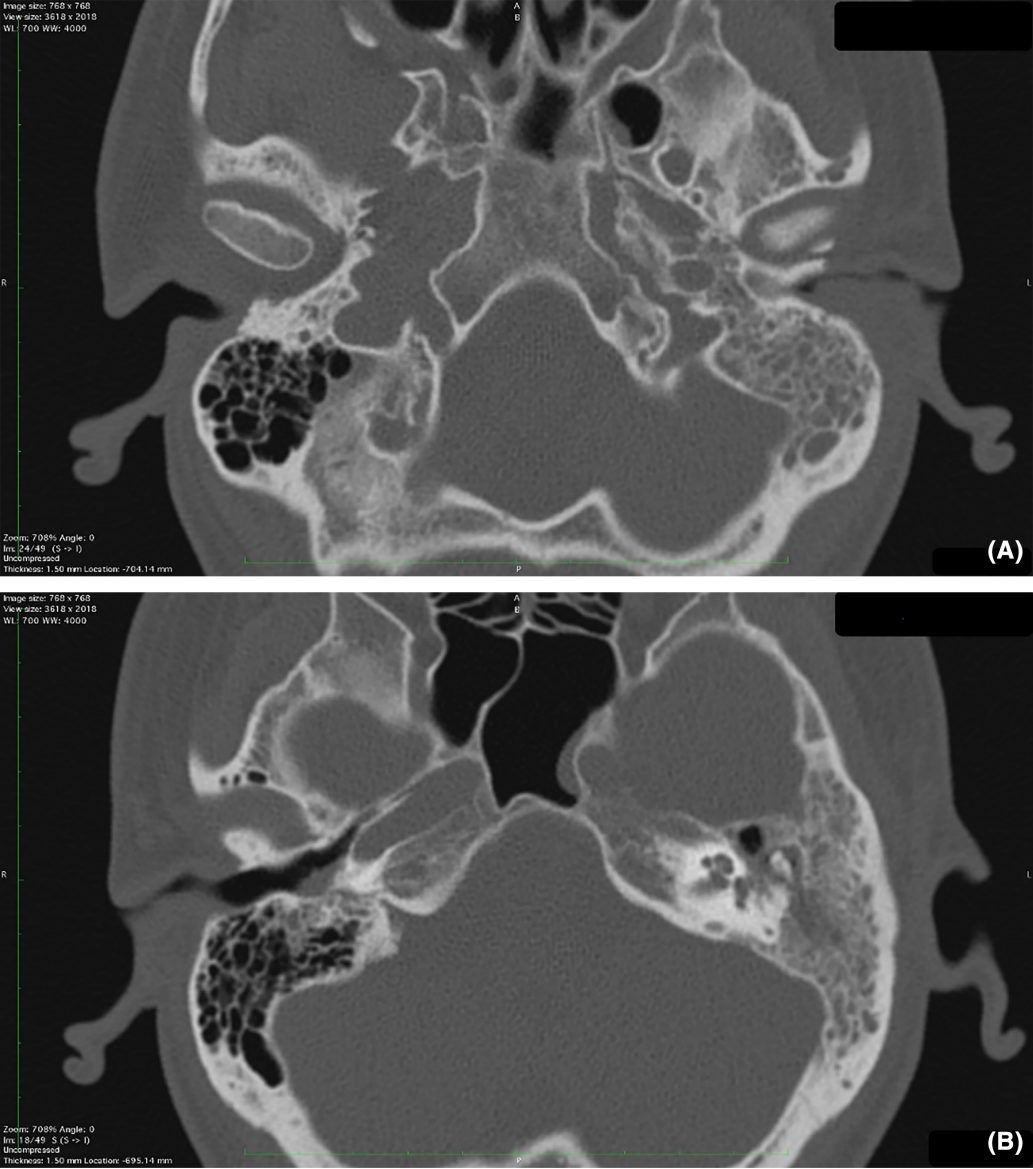
Head computed tomography. Computed tomography reveals a complete homogenous left‐sided and partial right‐sided opacification of the mastoid cells and the middle ear with a relatively intact bony structure. A significant narrowing of the left external auditory canal (A) is shown, compared to the right external auditory canal (B).

**FIGURE 3:**
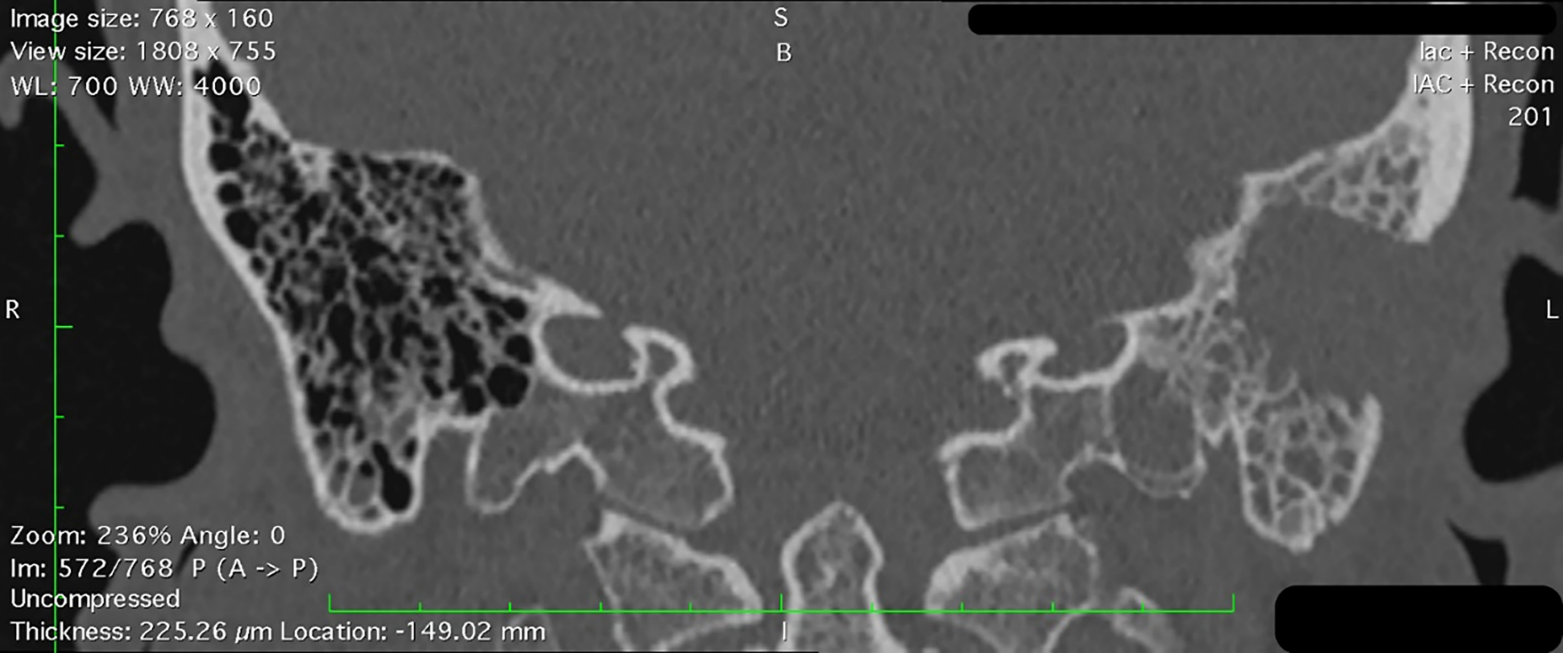
Head computed tomography, post‐mastoidectomy. Computed tomography reveals persistent opacification in the remaining mastoid cells on the left and partial opacification of the right mastoid cells.

A computed tomography (CT) scan was conducted (Figure 2), revealing a narrowed left external auditory canal and nonspecific bilateral opacification of the middle ear cavities and the mastoid cells, which was particularly notable on the left side. The mastoid structure appeared intact, with no apparent areas of bone destruction and distinct masses. Taking into account the clinical presentation and imaging findings, a provisional diagnosis of acute bilateral otomastoiditis was made. Due to progressive worsening of the symptoms and refractoriness to conservative therapy, a left‐sided mastoidectomy was performed (Figure 3). On visual inspection, the mastoid fragment contained no soft tissue.

**FIGURE 4:**
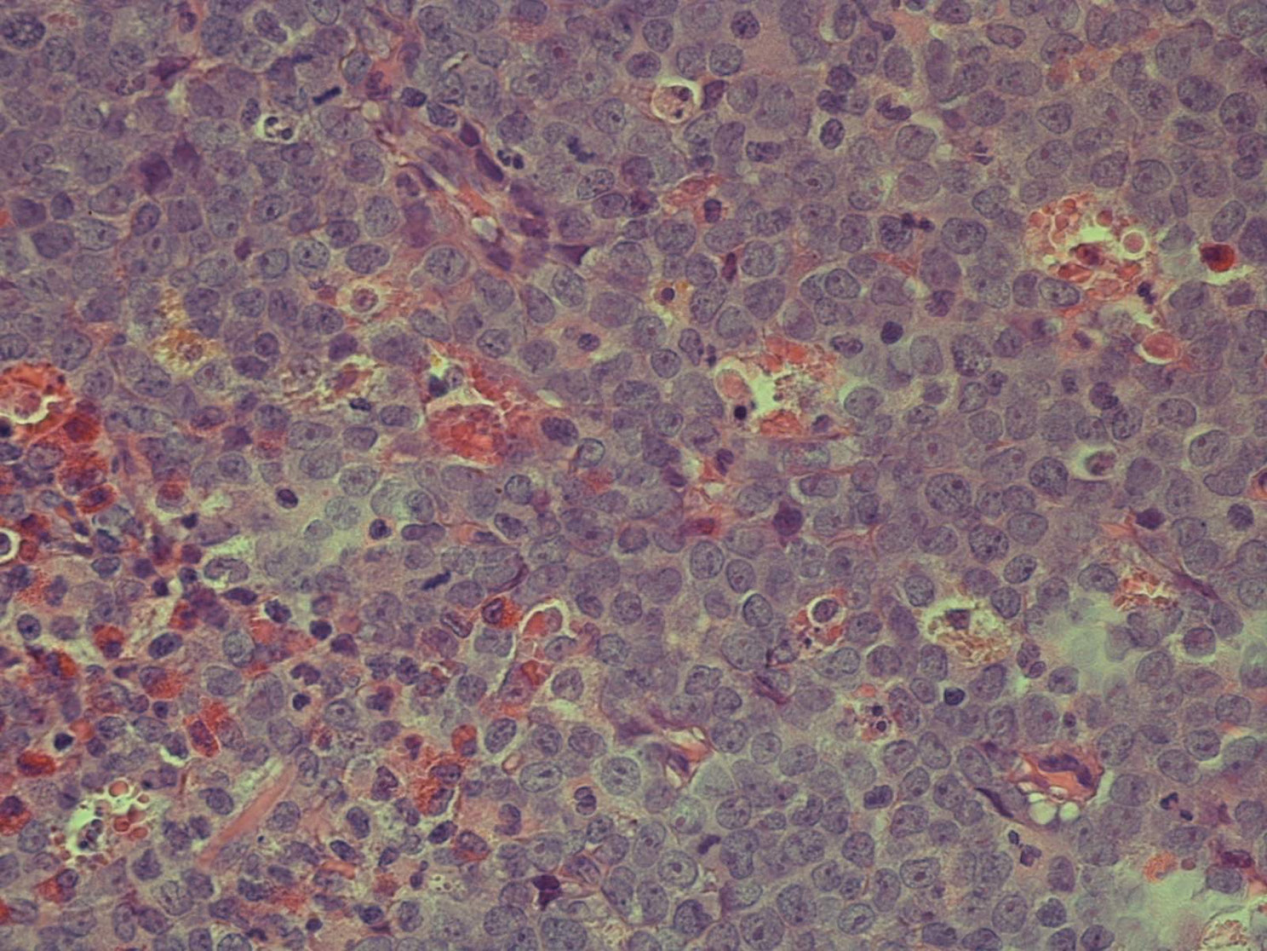
Histology of the left mastoid bone. Histologic evaluation shows diffuse infiltration with medium‐ and large‐sized cells with irregularly shaped nuclei and scant cytoplasm. Subsequent immunohistochemistry showed positive staining for myeloperoxidase, CD34, TdT, CD117, and CD68, indicative for the presence of myeloblasts.

The subsequent pathological review of the mastoid structure demonstrated extensive effacement with a homogeneous population of atypical cells characterized by irregular nuclei, fine chromatin, prominent nucleoli, and scant cytoplasm (Figure 4). An initial diagnosis of Burkitt's lymphoma was considered; however, further immunohistochemistry profiling showed positive staining for myeloperoxidase, CD34, TdT, CD117, CD68, and negative staining for CD3, CD20, PAX5, ALK, and Desmin. These results were consistent with the presence of blast cells of the myeloid lineage. Importantly, these findings were at odds with the absence of leukemic cells in the blood, bone marrow, and CSF. A final diagnosis of relapsed AML presenting as an isolated myeloid sarcoma of the temporal bone was established.

## 4 OUTCOME AND FOLLOW‐UP

**FIGURE 5:**
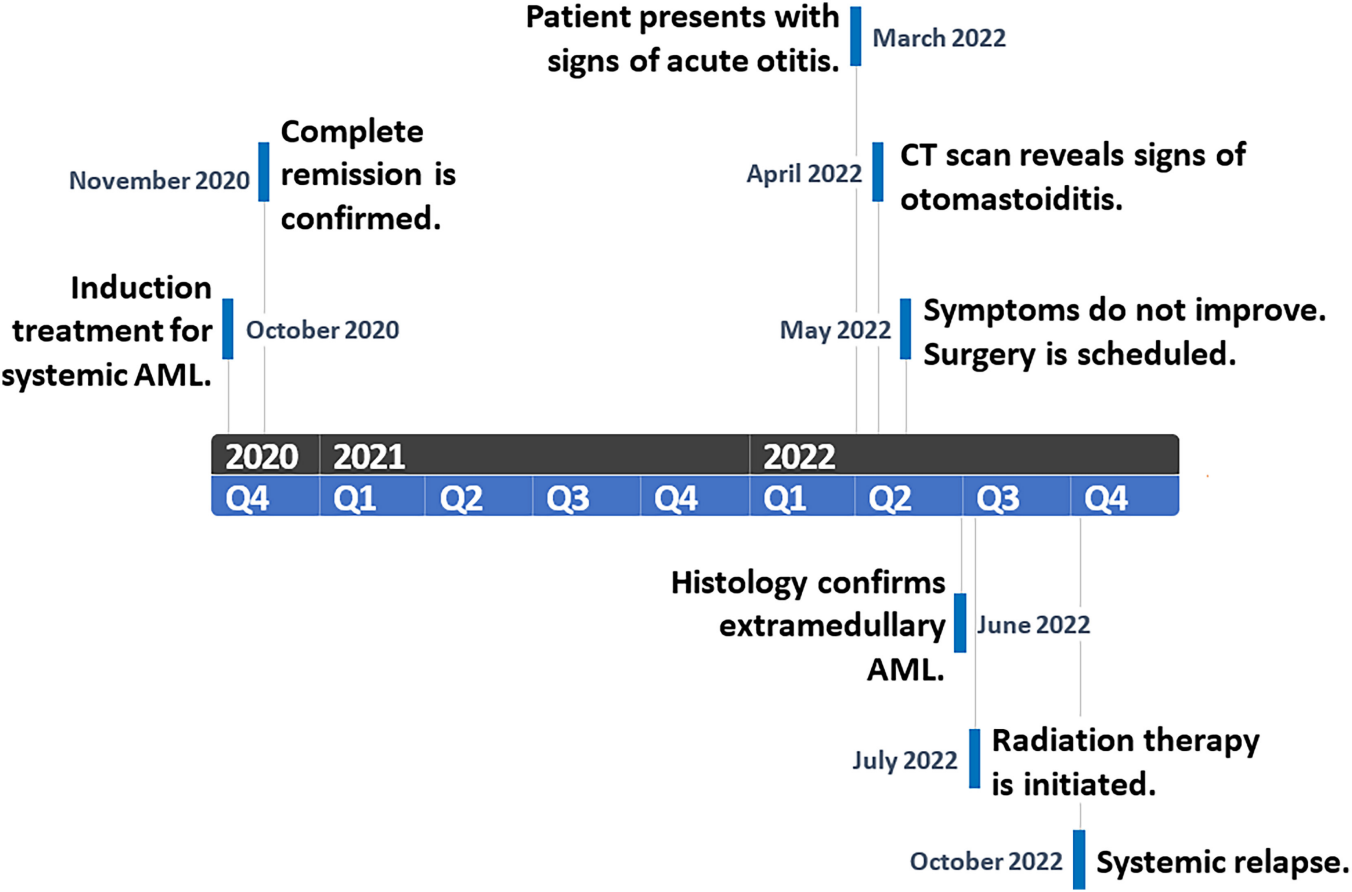
Event timeline.

Following the surgical intervention, the patient made a full recovery with significant symptom relief. Since the initial presentation, neither the CBC nor the bone marrow examination showed signs of leukemic involvement. As such, combined treatment with local radiation (28 Gy) and intrathecal chemotherapy with cytarabine, methotrexate and dexamethasone was attempted. However, by the end of radiation therapy, the patient presented once again with fever and progressive weakness (the event timeline is shown in Figure 5). Subsequent bone marrow aspiration confirmed a systemic relapse with 56% blast cells. Reinduction was attempted using the previously successful induction regimen; however, after two cycles, the patient failed to achieve remission.

## 5 DISCUSSION

Extramedullary AML, also known as myeloid sarcoma, is characterized by the infiltration of blast cells into normal tissues, as confirmed by histological examination.^1^ MS may develop either de novo or concomitantly with systemic AML. In some cases, it may precede systemic disease by several months or serve as the initial manifestation of relapsed AML.^3^ The most commonly affected sites are other hematopoietic organs (such as the liver, spleen, and lymph nodes), followed by skin and gingivae.^4^ Localized bone involvement, particularly in the temporal bone, is rare and has only been described in a few case studies. The underreporting of such cases could be a common issue that may be attributed to the diagnostic challenges associated with distinguishing myeloid sarcoma from other conditions, with misdiagnosis rates reported as high as 47% in recent studies.^6^

In cases similar to the one presented, several factors can contribute to an erroneous diagnosis. Patients with leukemia are prone to developing otologic symptoms, and acute leukemia patients are particularly susceptible to bacterial, fungal, or viral infections affecting the external, middle, or inner ear.^7^ Temporal bone involvement, specifically, may often manifest as a triad of otalgia, hearing loss, and facial paralysis due to facial nerve involvement.^8^, ^9^, ^10^ Progressive disease can also result in extensive bone erosions and bacterial superinfections, contributing to the development of acute otomastoiditis.^11^ Still, these symptoms are nonspecific. In our patient, considering the prolonged hematologic remission with no signs of systemic disease or central nervous system involvement, a provisional diagnosis of acute otitis media and externa was made.

Unsurprisingly, the subsequent conservative treatment proved ineffective. In some cases, a CT or magnetic resonance imaging (MRI) scan can aid in establishing the diagnosis. Specifically, extramedullary involvement could present as a well‐defined, solid mass, with frequent invasion of adjacent bony structures.^12^ However, imaging features are variable, site‐dependent, and may be indistinguishable from other, more common malignancies, namely lymphoma.^12^ Therefore, given a patient's history, even nonspecific opacification should be considered as potential evidence of neoplastic invasion mimicking a benign condition (such as otomastoiditis). Although PET/CT has demonstrated high sensitivity and specificity in identifying myeloid sarcoma, its results are heterogenous when it comes to concurrent bone marrow involvement,^13^ and interpretation can be even more challenging in cases of extramedullary bone disease and inflammatory complications. As such, imaging features can be insufficient for distinguishing not only between various neoplasms, but also nonmalignant disorders.

A biopsy of the lesion appears to be the only definitive method for confirming a case of extramedullary AML. Despite this, invasive procedures are frequently avoided or not feasible owing to certain frequent comorbidities, such as thrombocytopenia. As a consequence, the reported prevalence of myeloid sarcoma has previously varied from 2.5% to 9.1%.^2^ In contrast to this, a recent PET/CT‐based study revealed a prevalence of 22%.^13^ Likewise, another large‐scale study showed an overall incidence of extramedullary disease of 23.7%.^4^ Limited and challenging access to affected sites (including the most typical ones, such as liver and spleen) likely contributes to the underestimation in former reports, including cases involving the temporal bone. Finally, when present, extramedullary disease is generally considered a negative prognostic factor.^2^, ^14^, ^15^ Specifically, the site and pattern of leukemic involvement appear to be important for patient outcomes.^4^, ^16^ For instance, patients with leukemia cutis showed a significantly inferior overall survival when compared to other sites of disease (median of 5.7 months vs. 21.9 months, respectively).^16^ Interestingly, rare areas of involvement, including the bones, may be associated with better survival rates.^4^

Current management of AML involves standard multi‐agent chemotherapy with the possible addition of targeted therapy based on risk stratification and mutational profiling.^17^ There is a lack of prospective clinical trials, and treatment of extramedullary disease, specifically, remains controversial. However, the onset of isolated extramedullary relapse frequently heralds a bone marrow relapse with a mean interval of around 7 months.^18^ Accordingly, current recommendations for MS are based mainly on existing AML protocols, with or without the addition of radiation therapy and surgery.^2^, ^17^ In the present case, local treatment was attempted initially. Radiation therapy has shown excellent response rates (91%–97%) and local disease control, with a median progression‐free survival of 11 months.^19^, ^20^, ^21^ Cases of isolated temporal bone relapse with prolonged responses after radiation therapy have also previously been reported.^8^ Regardless, survival is poor even among those achieving clinical remission with local therapy, and the majority are likely to relapse.^22^ In the current case, systemic relapse occurred soon after the end of radiation therapy, possibly due to delayed recognition of the local relapse, and subsequent chemotherapy failed to achieve CR.

Improving management strategies and outcomes in myeloid sarcoma may involve novel therapy and targeting specific molecular alterations, similarly to systemic acute myeloid leukemia. However, genetic events underlying extramedullary disease are still not well understood and remain controversial.^15^ Potentially targetable mutations commonly encountered in AML have been associated with extramedullary disease in several recent next‐generation sequencing (NGS) studies.^15^, ^16^ In line with this, a study by Ball et al. demonstrated a complete response in three out of four patients treated with IDH inhibitors based on on‐site next‐generation sequencing of the MS tumor.^16^ Furthermore, significant discordance has been observed between the molecular profiles of the myeloid sarcoma tumors and concurrent bone marrow disease.^16^, ^23^ In one study, up to one‐third of cases showed molecular discordance, with the majority revealing discordance in prognostically important or potentially targetable alterations.^23^ Therefore, while not available at the time at our center, biopsy, followed by mutational analysis is crucial for optimizing future therapy of EM AML. Nonetheless, due to the low prevalence and varying presentation of myeloid sarcoma, our understanding of optimal management strategies is limited, and more prospective trials are necessary to evaluate the novel treatment approaches for extramedullary AML.

## 6 CONCLUSIONS

Extramedullary acute myeloid leukemia is a diagnostic challenge and can often mimic other disorders, presenting with non‐specific clinical and imaging findings. It is crucial to maintain a high level of suspicion, particularly in patients with a history of neoplastic disease. Biopsy, followed by immunophenotyping and molecular analyses, should ideally be performed in every patient with myeloid sarcoma. This is essential for an accurate differential diagnosis, but may also aid in proper risk stratification and optimization of treatment strategies for extramedullary disease.

## AUTHOR CONTRIBUTIONS

**Ivan Negara:** Conceptualization; writing – original draft; writing – review and editing. **Inga Chemencedji:** Investigation. **Natalia Dobrovolschi:** Investigation. **Natalia Sporis:** Supervision; validation. **Sanda Buruiana:** Supervision; validation; writing – original draft. **Igor Vinogradov:** Investigation; supervision; validation; writing – review and editing.

## FUNDING INFORMATION

Not applicable.

## CONFLICT OF INTEREST STATEMENT

The authors declare no conflicts of interest. All coauthors have seen and agreed with the contents of the article. We certify that the submission is original work and is not under review for any other publication.

## CONSENT

Written informed consent was obtained from the patient to publish this report in accordance with the journal's patient consent policy.

## Data Availability

The data that support the findings of this paper are available from the corresponding author upon reasonable request.

## References

[1] Swerdlow SH , Campo E , Harris NL , et al. WHO Classification of Tumours of Haematopoietic and Lymphoid Tissues. 4th ed. International Agency for Research on Cancer; 2017.

[2] Bakst RL , Tallman MS , Douer D , Yahalom J . How I treat extramedullary acute myeloid leukemia. Blood. 2011;118(14):3785‐3793. doi:10.1182/blood-2011-04-347229 21795742

[3] Pileri SA , Ascani S , Cox MC , et al. Myeloid sarcoma: clinico‐pathologic, phenotypic and cytogenetic analysis of 92 adult patients. Leukemia. 2007;21(2):340‐350. doi:10.1038/sj.leu.2404491 17170724

[4] Ganzel C , Manola J , Douer D , et al. Extramedullary disease in adult acute myeloid leukemia is common but lacks independent significance: analysis of patients in ECOG‐ACRIN cancer research group trials, 1980‐2008. JCO. 2016;34(29):3544‐3553. doi:10.1200/JCO.2016.67.5892 PMC5074349 27573652

[5] Noh BW , Park SW , Chun JE , Kim JH , Kim HJ , Lim MK . Granulocytic sarcoma in the head and neck: CT and MR imaging findings. Clin Exp Otorhinolaryngol. 2009;2(2):66‐71. doi:10.3342/ceo.2009.2.2.66 19565030 PMC2702726

[6] Yilmaz AF , Saydam G , Sahin F , Baran Y . Granulocytic sarcoma: a systematic review. Am J Blood Res. 2013;3(4):265‐270. 24396704 PMC3875275

[7] Andrès E , Kurtz JE , Maloisel F , Dufour P . Otological manifestations of acute leukaemia: report of two cases and review of literature. Clin Lab Haematol. 2001;23(1):57‐60. doi:10.1046/j.1365-2257.2001.00358.x 11422232

[8] Jung DJ , Lee HJ , Kwak JH , Lee KY . A case of myeloid sarcoma mimicking Otomastoiditis with Retroauricular abscess. J Clin Otolaryngol Head Neck Surg. 2019;30(1):77‐82. doi:10.35420/jcohns.2019.30.1.77

[9] Murakami M , Uno T , Nakaguchi H , et al. Isolated recurrence of intracranial and temporal bone myeloid sarcoma—case report. Neurol Med Chir (Tokyo). 2011;51(12):850‐854. doi:10.2176/nmc.51.850 22198109

[10] Zheng HD , Abdel‐Aty Y , Taylor C , et al. Myeloid sarcoma of the temporal bone: a unique cause of hearing loss, otalgia, and facial nerve weakness. Otol Neurotol. 2022;43(4):e435‐e441. doi:10.1097/MAO.0000000000003478 35120076

[11] Almadori G , Del Ninno M , Cadoni G , Di Mario A , Ottaviani F . Facial nerve paralysis in acute otomastoiditis as presenting symptom of FAB M2, T8;21 leukemic relapse. Case report and review of the literature. Int J Pediatr Otorhinolaryngol. 1996;36(1):45‐52. doi:10.1016/0165-5876(95)01323-7 8803691

[12] Singh A , Kumar P , Chandrashekhara SH , Kumar A . Unravelling chloroma: review of imaging findings. Br J Radiol. 2017;90(1075):20160710. doi:10.1259/bjr.20160710 28445074 PMC5594979

[13] Stölzel F , Lüer T , Löck S , et al. The prevalence of extramedullary acute myeloid leukemia detected by 18FDG‐PET/CT: final results from the prospective PETAML trial. Haematologica. 2020;105(6):1552‐1558. doi:10.3324/haematol.2019.223032 31467130 PMC7271590

[14] Cornelissen JJ , Gratwohl A , Schlenk RF , et al. The European LeukemiaNet AML working party consensus statement on allogeneic HSCT for patients with AML in remission: an integrated‐risk adapted approach. Nat Rev Clin Oncol. 2012;9(10):579‐590. doi:10.1038/nrclinonc.2012.150 22949046

[15] Eckardt JN , Stölzel F , Kunadt D , et al. Molecular profiling and clinical implications of patients with acute myeloid leukemia and extramedullary manifestations. J Hematol Oncol. 2022;15(1):60. doi:10.1186/s13045-022-01267-7 35562747 PMC9107142

[16] Ball S , Knepper TC , Deutsch YE , et al. Molecular annotation of extramedullary acute myeloid leukemia identifies high prevalence of targetable mutations. Cancer. 2022;128(21):3880‐3887. doi:10.1002/cncr.34459 36107670

[17] Heuser M , Ofran Y , Boissel N , et al. Acute myeloid leukaemia in adult patients: ESMO clinical practice guidelines for diagnosis, treatment and follow‐up†. Ann Oncol. 2020;31(6):697‐712. doi:10.1016/j.annonc.2020.02.018 32171751

[18] Byrd JC , Weiss RB . Recurrent granulocytic sarcoma. An unusual variation of acute myelogenous leukemia associated with 8;21 chromosomal translocation and blast expression of the neural cell adhesion molecule. Cancer. 1994;73(8):2107‐2112. 7512442 10.1002/1097-0142(19940415)73:8<2107::aid-cncr2820730815>3.0.co;2-w

[19] Bakst R , Wolden S , Yahalom J . Radiation therapy for chloroma (granulocytic sarcoma). Int J Radiat Oncol Biol Phys. 2012;82(5):1816‐1822. doi:10.1016/j.ijrobp.2011.02.057 21962486 PMC5045241

[20] Oertel M , Elsayad K , Haverkamp U , Stelljes M , Eich HT . Radiotherapy for extramedullary leukaemic manifestation (Chloroma). Strahlenther Onkol. 2018;194(2):164‐173. doi:10.1007/s00066-017-1236-4 29147841

[21] Hall MD , Chen YJ , Schultheiss TE , Pezner RD , Stein AS , Wong JYC . Treatment outcomes for patients with chloroma receiving radiation therapy. J Med Imaging Radiat Oncol. 2014;58(4):523‐527. doi:10.1111/1754-9485.12172 24649928

[22] Cunningham I , Kohno B . 18FDG‐PET/CT: 21st century approach to leukemic tumors in 124 cases. Am J Hematol. 2016;91(4):379‐384. doi:10.1002/ajh.24287 26718745

[23] Werstein B , Dunlap J , Cascio MJ , et al. Molecular discordance between myeloid sarcomas and concurrent bone marrows occurs in actionable genes and is associated with worse overall survival. J Mol Diagn. 2020;22(3):338‐345. doi:10.1016/j.jmoldx.2019.11.004 31866570

